# The Role of Insects in Agri-Food Sustainability: Taking Advantage of Ecosystem Services to Achieve Integrated Insect Management

**DOI:** 10.3390/insects16080866

**Published:** 2025-08-20

**Authors:** Karol B. Barragán-Fonseca, Julio Esteban Ortiz, Juan D. García-Arteaga, David Giron

**Affiliations:** 1 Centre for Terrestrial Arthropod Research (CINAT), Faculty of Veterinary Medicine and Animal Science, Universidad Nacional de Colombia, Bogotá D.C. 11001, Colombia; 2 Faculty of Medicine, Universidad Nacional de Colombia, Bogotá D.C. 11001, Colombia; judgarciaar@unal.edu.co; 3 Institut de Recherche sur la Biologie de l’Insecte (IRBI) UMR7261, Centre National de la Recherche Scientifique, Université de Tours, 37000 Tours, France; directeur.irbi@univ-tours.fr

**Keywords:** integrated insect management, bioeconomy, insect-based innovation

## Abstract

The global agrifood system faces complex challenges related to biodiversity loss, food insecurity, and environmental degradation. Insects, as providers of multiple ecosystem services, offer a largely untapped potential to contribute to sustainability. This article introduces a conceptual framework for understanding the socio-ecological roles (SER) of insects, encompassing not only provisioning, regulating, supporting, and cultural services, but also the risks and challenges associated with their use and management. Through an interdisciplinary synthesis, we analyse four complementary insect management strategies—conservation, pest and vector control, wild insect gathering, and insect farming—and examine the relationship of these strategies to ecosystem services and stakeholder dynamics, as well as the need for policies regulating insect use and conservation. We argue that combining these strategies rather than treating them as isolated approaches enhances the capacity to optimize insect contributions while mitigating trade-offs. This holistic perspective contributes to a systemic, inclusive, evidence-based foundation for decision-making regarding the use of insect biodiversity to achieve resilient, sustainable agrifood systems.

## Simple Summary

Insects and their derivatives are increasingly valued for practical uses in food, feed, waste management, and cultural applications, as well as for essential roles in ecosystems. These contributions fall within the four main categories of ecosystem services: provisioning, regulating, supporting, and cultural. However, ecological imbalances or poor management can turn them into challenges such as disease transmission or crop damage. This paper presents a framework that integrates four insect management areas—conservation, pest and vector control, wild gathering, and farming—to enhance benefits and reduce risks. Linking these domains with the services insects provide and the people involved offers an integrated pathway to protect biodiversity, ensure food safety, and promote the sustainable use of insects in agri-food systems.

## 1. Introduction

Biodiversity is essential for the functioning of agroecosystems and for the stability of food production. Nevertheless, conventional food systems have ironically become one of the major drivers of biodiversity loss worldwide. The current agrifood system exerts increasing pressure on finite resources and intensifies competition over land to produce food, feed, and fuel [1,2], with approximately 30–40% of all food produced going to waste along the production chain [3]. To conserve biodiversity and reduce human impact on ecosystems, it is imperative to transition from a model of a single linear food system to one composed of multiple circular food systems that incorporates more sustainable nutrient sources and more effectively recycles waste [4,5].

Understanding local biodiversity and its ecological functioning, as well as technological innovation, is essential to supporting effective ecosystem management [6,7]. This in turn may contribute to designing locally adapted biodiversity-based food systems that are sustainable and resilient, while also conserving species and habitats [8,9]. In this context, insects provide a powerful yet underexplored tool for sustainability. Their exceptional diversity contributes to the health, complexity, and resilience of ecosystems globally [10]. With over 5.5 million species estimated worldwide—at least 1 million of which have been scientifically described [11,12], insects have evolved to occupy virtually every ecological niche [10,13]. Furthermore, insects significantly contribute to all four categories of ecosystem services (ES) as defined by the Millennium Ecosystem Assessment (MEA): provisioning, regulating, supporting, and cultural services [14,15]. These contributions have prompted a growing interest in insects as agents to improve agrifood sustainability [16], ensure food security, and address multiple United Nations Sustainable Development Goals (SDGs), such as 2, 12, 13, and 15 [17,18].

Despite the potential ecosystem benefits of insects, traditional classifications of insect ES and disservices tend to reflect anthropocentric perspectives, categorizing insects according to their perceived utility or threat to human systems. While the MEA framework may be useful, it does not allow for capturing the full complexity of insect–human–ecosystem interactions. For this reason, we introduce the concept of insect socio-ecological roles (SER)—an integrative perspective which transcends the narrow binary evaluation of benefit vs. harm [19]. This concept acknowledges insects as agents with multiple context-dependent roles, and therefore that differentiated strategies are necessary for their management.

The SER approach to insect management emphasizes not only the beneficial services insects provide but also the ecological and social challenges they may pose. These challenges are not inherent to the insects themselves but rather arise due to ecological imbalances, environmental degradation, and/or inadequate management. We argue that understanding insects’ socio-ecological roles from a systemic multidimensional perspective may allow for developing urgently needed sustainable insect management strategies. Despite their critical functions in nature, insects are generally managed in a fragmented manner. Policymakers, researchers, and farmers often work in isolation, missing opportunities for effective synergetic action [19]. Given accelerating climate change and biodiversity loss, integrating biological knowledge into stakeholder engagement and cross-sectoral governance is no longer an option, but rather a necessity.

Recognizing insects as providers of SER in both natural ecosystems and under human management provides new opportunities to address environmental, technical, and societal challenges in agrifood systems while contributing to sustainability goals. This article examines how insects act as nature-based solutions to sustainability challenges in agrifood systems through regulating, supporting, provisioning, and cultural functions. It is organized into three sections, each of which examines a guiding question: Section 2—How do insects contribute to agrifood systems, and what risks arise when their functions are disrupted? Section 3—What are the principal strategies for insect management, and how are they linked to provision of ecosystem services, roles of different stakeholders, and specific governance needs? Section 4—How may these strategies be strategically combined to enhance sustainability and optimize insect contributions in different contexts? By addressing these questions, we propose a critical framework connecting ecosystem services, management strategies, and decision-making needs which may allow for transitioning to the sustainable uses of insect biodiversity which are inclusive of different actors and types of knowledge.

## 2. Conceptual Approach

This article presents an integrative conceptual framework for evaluating the SER of insects in agrifood systems. We combined an extensive literature review with interdisciplinary insights from insect ecology, sustainable agriculture, and wildlife management, with the goal of exploring and summarizing key areas of knowledge that contribute to a multidimensional understanding of insects’ roles. Specifically, we aimed to (a) identify principal ES provided by insects; (b) explore socio-ecological challenges linked to insects’ roles; (c) analyse conceptual frameworks addressing ES and their relevance to insect management; and (d) outline strategic insect management approaches that optimize insect contributions while mitigating associated risks.

### 2.1. Integrative Literature Exploration

We conducted an extensive literature exploration in Scopus, Web of Science, and Google Scholar using a combination of key words, including “ecosystem services”, “socio-ecological roles”, “insect management”, “agriculture”, “agrifood”, “sustainability”, and “biodiversity”, focusing on peer-reviewed articles, conceptual frameworks, case studies, and reviews addressing insects’ contributions to ES within agrifood systems. Special attention was given to the MEA categories—provisioning, regulating, supporting, and cultural services [14,20,21]—as a foundational framework for mapping insect roles. Additionally, we considered the literature that highlighted disservices and risks associated with insects. This exploratory review was complemented by the authors’ interdisciplinary expertise in insect farming, conservation, sustainable use of wildlife, entomology, and the One Health concept (i.e., interconnectedness among human, animal, and environmental health), which allowed for exploring conceptual gaps, context-specific dynamics—such as variations in ecological conditions, cultural practices, and regulatory frameworks—and previously overlooked opportunities for integrated insect management.

### 2.2. Framework Development and Core Management Areas

The conceptual framework proposed in this article expands on conventional ecosystem service categories upon addressing challenges—or negative or conflictive roles that insects may play in agrifood contexts. This acknowledges the complex nature of the ES of many insect species and highlights the need to balance benefits and risks upon designing insect management strategies.

Rather than treating insect conservation, control, production, and other types of use as separate topics, we propose an integrated understanding of their SER that reflects their ecological complexity and management potential. Based on the literature review and the authors’ field experience, we identified four core areas of insect management that reflect differentiated approaches to insect roles in agrifood systems: conservation management, pest and vector control, wild insect gathering, and insect farming. Each is carried out in specific socio-ecological contexts, has specific objectives, and should be taken into account upon formulating sustainable policy development and research agendas. Together, they constitute the foundation of the integrated insect management framework, which is further discussed below.

## 3. A Socio-Ecological Perspective of Insect Contributions Considering Both Ecosystem Services and Challenges

Insects are one of the most taxonomically and functionally diverse taxa on Earth and make up a substantial proportion of global biomass [15]. While insects play essential roles in both natural and managed ecosystems, dominant ecosystem service frameworks often narrowly focus on human benefits [22,23]. This anthropocentric perspective tends to overlook the multidimensionality of their contributions, including ecological benefits as well as potential disservices and ecological risks. To contribute to a more holistic, context-sensitive perspective of insect management, we introduce the concept of SER, which encompasses both beneficial and adverse contributions of insects to agrifood systems. This framework includes the four MEA categories as well as a complementary dimension, namely challenges—referring to insect-related risks, disservices, and tensions that emerge when insects’ ecological roles conflict with ecosystem stability and/or human needs, including their socio-technical systems.

**Figure 1:**
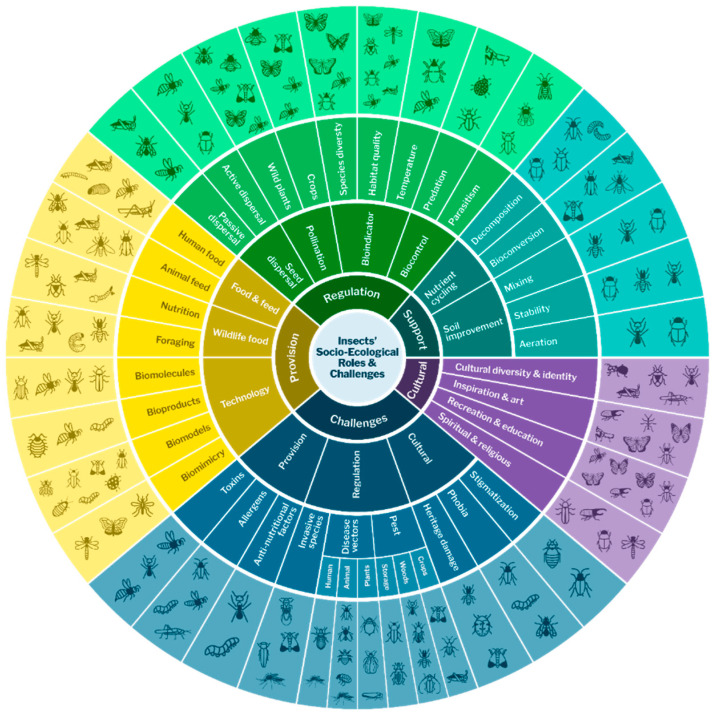
Socio-ecological roles and challenges provided by insects on a global level.

Figure 1 provides a visual summary of insects’ SER, organised according to the four main ES categories—provisioning, regulation, support, and cultural services, including different types of roles within each of these categories. It also indicates potential challenges—or disservices—for three of these categories (provisioning, regulation, and cultural services). Insect icons surrounding the framework illustrate species commonly responsible for each role or risk. This diagram illustrates the idea that insect contributions and challenges are context-dependent and therefore call for local management approaches that address their multifunctionality as both allies and potential threats.

### 3.1. Regulating Roles: Ecological Balance and Regulatory Challenges in Agrifood Systems

Insects provide essential regulating functions that sustain the ecological balance of agrifood systems. These SER include pollination [24,25,26], seed dispersal [27], bioindication [28,29,30], and biological control [31], which are critical for maintaining agroecosystem resilience, reducing chemical inputs, and supporting biodiversity [32,33]. However, when these functions are disrupted or mismanaged, they may generate significant challenges, including pest outbreaks, biological invasions, and vector-borne diseases [34,35,36]. Understanding these dynamics is essential for designing context-specific insect management strategies that enhance regulation while minimizing risks.

Pollination and seed dispersal are two of the most visible and beneficial regulatory roles of insects [24]. Approximately 70% of plants rely on insect pollinators, and 30% of these are directly involved in agriculture [25]. However, decline in pollinators as a result of pesticide use, habitat loss, and agro-industrial practices threatens both biodiversity and food production [26]. While honeybees (*Apis mellifera*) are the most widely recognized pollinators, native pollinators including solitary bees, bumblebees, butterflies, beetles, moths, flies, and even mosquitoes play equally crucial roles, particularly in biodiverse tropical regions [25]. Ants and dung beetles also contribute to ecosystem regulation through seed dispersal, thereby supporting forest regeneration and maintenance of plant diversity [27].

Insects serve as bioindicators of ecosystem health due to their sensitivity to environmental change and rapid life cycles [28]. For example, accumulation of trace metals in *Coenonympha pamphilus* (small heath butterfly) indicates soil contamination in industrial zones [29], while genotoxic effects of particulate matter ≤10 µm (PM_10_) in urban settings may be found in the bodies of *Pieris brassicae* (large white butterfly) [30]. Monitoring changes in insect diversity, abundance, and behaviour thus provides early warning signs of ecosystem stress [31]. The presence of certain insect species may also indicate environmental degradation, for instance when pest species proliferate, or disease vectors become more widespread.

Biological control is another key regulatory service [32]. Predatory and parasitic insects, such as ladybugs, lacewings, mantises, hoverflies, and Trichogramma wasps, are essential allies in reducing pest populations [33,34,35]. Biological control is increasingly being integrated into agroecological systems to minimize pesticide use [36]. However, these functions are not without risks: insects introduced into non-native ecosystems may become invasive, disrupting ecological networks by outcompeting local species, altering habitats, or transmitting new diseases [37,38]. Their rapid spread is often exacerbated by human activity, including commerce, land-use change, and climate shifts. Such imbalances may reduce agricultural productivity, increase economic costs, and weaken ecological stability, particularly in areas lacking sustainable pest management systems [28,39,40]. Moreover, failure to adapt control techniques—such as the release of natural predators or sterile insects—to the local context may disrupt food webs and prove ineffective where invasive pests are already dominant.

Pest insects continue to pose critical threats to global food security. Although they make up only a small fraction of insect biodiversity, 20–30 species cause over 20% of global crop loss, valued at over USD 470 billion annually [41]. Species including *Spodoptera frugiperda* (fall armyworm), *Aphis gossypii* (cotton aphid), and *Tribolium castaneum* (red flour beetle) commonly damage both crops in the field as well as post-harvest food systems [42], with disproportionately severe consequences for low-income and otherwise socioeconomically vulnerable populations [38]. Recurrent outbreaks reflect systemic breakdown in regulatory balance and highlight the need for ecologically informed, socially grounded responses. Insects are also key disease vectors. Mosquitoes of the genera *Anopheles*, *Aedes*, and *Culex* transmit pathogens such as malaria, dengue, Zika, and West Nile virus, with profound impacts on human health and economies [10,43]. Similarly, hemipteran insects—including aphids, thrips, whiteflies, and leafhoppers—act as vectors of plant viruses, reducing crop resilience and productivity [44,45,46].

Viewed through a socio-ecological lens, regulatory functions are some of insects’ most impactful and multidimensional roles. They simultaneously embody potential for ecological equilibrium as well as risk of cascading system failures. Recognizing this duality is critical for shifting from reactive control measures to adaptive, ecosystem-based management strategies. This includes not only pollinator protection and invasive species control but also enhanced ecological pest regulation and disease vector surveillance. Managing these regulatory SER may effectively strengthen the resilience of agroecosystems, reduce their vulnerability to environmental disturbances and biological threats, and position insects as sentinels contributing to broader socio-ecological health.

### 3.2. Provisioning Roles (Food, Feed, and Biomolecules) and Their Associated Risks

Insects have long been recognized as fundamental components of food webs, serving as prey for a wide array of wildlife, including birds, mammals, amphibians, reptiles, and fish, particularly in tropical and freshwater ecosystems where their abundance and diversity are greatest [10,23,47]. For example, caterpillars are a key protein source for canopy-dwelling birds and primates, while ants and termites are consumed by anteaters, monkeys, and kinkajous [48,49]. Aquatic insects such as nymphs of Odonata (dragonflies and damselflies) and larvae of Trichoptera (caddisflies) are essential to the diets of fish and amphibians, acting as pivotal links among trophic levels in freshwater trophic networks [50].

Beyond their ecological function as prey, insects are increasingly being cultivated and gathered in the wild for human consumption (entomophagy) and animal feed [51,52,53]. Over 2205 edible insect species have been scientifically documented [54], with entomophagy practiced in at least 128 countries, primarily among Indigenous peoples, smallholder communities, and hunter–gatherer societies throughout Africa, Asia, and the Americas [52,55,56]. Beetles are the most highly consumed insect order (31%), followed by caterpillars (18%), Hymenoptera (bees, ants, and wasps, 15%), and Orthoptera (crickets, grasshoppers, and locusts, 13%) [14,57]. Their nutritional profile, which includes high-quality proteins, lipids, and micronutrients, rivals and even exceeds that of conventional meats, making them a valuable resource for overcoming malnutrition and food insecurity [58]. Moreover, their efficient feed conversion rates and low environmental impact have propelled interest in incorporating them into livestock feed, particularly for poultry, fish, and pigs [59]. In recent years, advances in farming technologies have accelerated production of species including *Hermetia illucens* (black soldier fly), *Musca domestica* (housefly), *Tenebrio molitor* (yellow mealworm), *Acheta domesticus* (house cricket), and *Rhynchophorus* spp. (palm weevils), resulting in a growing insect-based bioeconomy [56,60,61,62].

This emerging sector has also catalysed a cascade of benefits through use of other insect products. Frass—the waste excreted by farmed insects—is increasingly being used as a biofertilizer with the potential of reducing reliance on synthetic inputs, improving soil health, and supporting existing circular farming systems [63]. Such multifunctional uses strengthen the case for insects as key actors in sustainable waste management and transition to circular economies. However, despite the promising potential of commercial insect farming, thus far it has been limited to fewer than ten species [64,65], raising concern regarding genetic bottlenecks and ecosystemic impacts of mass production [37].

Insects’ provisioning roles also have bio-industrial and technological applications, upon insects or their derivatives being used as biomodels, raw materials, and bioreactors [15,66,67]. Classic examples include the use of *Drosophila melanogaster* (fruit fly) in genetic research, *Bombyx mori* (silkworm) in silk production, and *Manduca sexta* (tobacco hornworm) for recombinant protein synthesis in biopharmaceuticals [68]. In medical research, insects provide a low-cost, ethically viable alternative to vertebrate models which is aligned with the 3R principle (reduce, reuse, and recycle) and enables high-throughput experimentation due to their short life cycles. In addition to whole organisms, insect cell lines—particularly from Lepidoptera—are used to produce industrial and pharmaceutical biomolecules [66,69], often with the aid of symbiotic bacteria, fungi, or viruses that reside inside insects [70,71,72].

Multiple insect bioproducts have long-held cultural significance and commercial uses, including as honey, silk, chitin, and carmine [73]. Chitin—one of the planet’s most abundant biopolymers—has notable antimicrobial and immunological properties, and silk by-products are currently being explored for use in cosmetic formulations and circular agro-industrial supply chains [74,75]. The structural features of insect cuticles and wings have inspired biomimetic technologies, including biosensors and energy storage devices, and bioluminescent compounds from Diptera offer innovative applications in pest control and environmental monitoring [76].

Despite these uses of insect products, insects’ provisioning roles are not exempt from critical challenges. Insects intended for feed and food may carry bioactive compounds, ranging from allergens and anti-nutritional factors to toxins, all of which pose risks to human and animal health [77,78]. For example, pan-allergenic proteins have been identified in 239 arthropod species, and may trigger anything from mild reactions to anaphylaxis [78]. Other compounds such as oxalates, tannins, alkaloids, phytates, and chitin may reduce nutrient bioavailability, while toxins synthesized or accumulated by insects (e.g., from plant secondary metabolites) may cause adverse effects upon ingestion [77]. Moreover, insect-based products may be contaminated by hazardous substances, including heavy metals, dioxins, pesticides, and veterinary medicines [79], particularly when insects have been reared on poorly regulated substrates. Even non-dietary contact with insects may cause negative health effects in humans and animals, including inflammatory responses of the skin or mucous membranes [80,81].

Through their provisioning SER, insects directly contribute to sustainability, innovation, and human well-being. They provide tangible resources—including food, feed, industrial and technological materials, and molecules for biotechnological applications, and have the potential to reshape agri-food systems. Nonetheless, making widespread use of their products requires considering other factors in addition to productivity and efficiency. Viewing insects’ provisioning roles from an SER perspective reveals critical dimensions of their use which are often overlooked: food safety, socio-environmental equity, cultural acceptance, and long-term agrifood system resilience. Therefore, advancing insect-based agri-food systems requires not only technical development but also inclusive regulatory frameworks, diversification beyond a few species, and policies grounded in the realities of each particular socio-ecological context.

### 3.3. Cultural Roles: Symbolic Power vs. Societal Tensions

The relationship between humans and insects extends far beyond ecological interactions; in all civilizations, it has been deeply embedded in cultural expressions, spiritual beliefs, and symbolic frameworks—including myths, rituals, and metaphors—that shape human–insect relationships in all cultures [10]. For millennia, insects have been present in human traditions, appearing in rituals, art, food, and storytelling. In many traditional communities, edible insects are not merely nutritional resources but also hold cultural significance, thereby contributing to collective well-being and identity [52]. For example, crickets and grasshoppers are celebrated not only for their role in dietary culture but also for their inclusion in cosmologies that form part of humanity’s intangible heritage and, as a result, in folklore—including songs and oral traditions [64,82]. Insects have inspired mythologies worldwide, with engravings and cave paintings depicting them dating back over 30,000 years [83].

The symbolism attached to insects is as diverse as the societies that have interacted with them. In Africa, termites are associated with fertility, abundance, and communication with ancestral spirits. In Europe, the honeybee has held a revered status in mythology, symbolizing order, industry, and sacred knowledge [53]. In North American indigenous traditions, the iconic migration of the monarch butterfly (*Danaus plexippus*) is a powerful metaphor for transformation and resilience in indigenous traditions [80]. In South America, leafcutter ants (*Atta* spp.) are not only edible resources; their sophisticated fungus gardens and social structures are revered and represented in spiritual narratives and ceremonial practices [84,85]. Such symbolism often emerges from the complex social behaviour of insects, particularly in the case of eusocial species such as ants, bees, and termites, which are perceived as metaphors of cooperation, resilience, and collective intelligence [15].

Relationships between humans and insects are also found in material culture. Insect products have long been associated with craftsmanship and status; for example, silk produced using domesticated moths and vibrant red dye made from cochineal (*Dactylopius coccus*) is used for clothing, food, and cosmetics. Insects are also recurrent subjects in art, literature, architecture, music, and popular culture, evoking fascination, fear, and admiration. They appear in stories and poems, album covers, and museum exhibits, challenging the boundaries between nature and imagination [15,82,86].

However, these cultural contributions coexist with negative perceptions, resulting in powerful tensions. Entomophobia, the intense fear of insects, and food neophobia, the rejection of unfamiliar foods, persist as cultural barriers to entomophagy while also inhibiting the implementation of broader insect conservation efforts [87]. These fears are often irrational, yet deeply ingrained, affecting not only individual diets but also national food policies. Entomophobia may provoke distress and avoidance of insects, reinforce negative stereotypes, and sever the emotional and symbolic connections necessary for insect conservation in general [88]. In fact, some authors suggest that indifference and aversion toward insects may be contributing to the reduced protection of insects and, in turn, their global decline [88]. Stigmatization resulting from aversion further compounds these issues. For example, cockroaches and bedbugs are often associated with poverty, poor hygiene, and marginalization. This socio-symbolic sense of contamination not only disincentivizes their protection but also reinforces social exclusion toward human communities that live in proximity to these insects [89]. Overcoming such stigma requires reframing insects not as pests or threats but as sentient participants in shared ecological world, as well as in our cultures.

Heritage also faces insect-related challenges. Termites, mites, and beetles such as *Anthrenus* spp. damage historical buildings, manuscripts, tapestries, and museum collections [90]. These impacts are often overlooked by mainstream conservation efforts, particularly when caused by non-native species, which—in the case of Europe—currently account for over half of all species threatening cultural heritage [91]. However, despite the direct connection between pest control and heritage preservation, these issues are rarely addressed in conjunction.

Cultural SER provide a unique opportunity for improving insect management by aligning management and public awareness efforts with local beliefs, traditions, and values. Understanding how insects are perceived—whether as symbols, taboos, or sources of pride—may guide context-specific insect management strategies that enhance acceptance, the participation of stakeholders in insect-related practices, and conservation. Rather than treating culture as a barrier, integrating it into SER-based approaches may contribute to unleashing social capital, reducing stigma, and fomenting inclusive, resilient agroecological transitions.

### 3.4. Supporting Roles: Foundations of Ecosystem Resilience

Insects’ supporting roles sustain ecosystem functions in ways that are often invisible but foundational to agroecosystem resilience. These roles include soil structuring, organic matter decomposition, nutrient cycling, and symbiotic interactions that enhance bioconversion. Beetles, ants, termites, and other insects significantly influence soil structure by aerating substrates and incorporating organic matter. For example, dung beetles not only bury animal faeces to later feed upon and use as nesting material but also improve water infiltration and reduce erosion through their tunnelling behaviour [10]. Similarly, ants and termites contribute to tilling the soil by transporting soil particles, providing aeration, and mixing minerals with organic matter [92].

Insects are also key agents in decomposition of organic matter and in nutrient cycling. Scarabaeidae dung beetles, including *Copris* spp. and *Onthophagus* spp., bury and decompose faecal matter; Silphidae beetles, including *Nicrophorus* spp., bury vertebrate carcasses; and Dermestidae, including *Dermestes* spp., scavenge animal remains, accelerating decomposition and nutrient release [93,94]. These functions are also fulfilled by aquatic insects, including Hydrophilidae (water scavenger beetles) and Dipteran larvae from Chironomidae, Culicidae, and other families, which break down submerged detritus in freshwater ecosystems [95].

In addition to passive decomposition, many insects simultaneously contribute to regulatory and provisioning SER through active bioconversion, which in turn contributes to circular economies by converting organic matter into protein-rich biomass and nutrient-rich fertilizer [96]. Successful bioconversion depends on several factors, including environmental conditions, species–substrate compatibility, and in some cases symbiotic microbial associations that aid digestion [97,98,99]. *M. domestica* and *H. illucens* are notably effective in decomposing food waste and manure, as well as reducing pathogens and odours [100,101]. In addition, *T. molitor* and *Galleria mellonella* (greater wax moth) have demonstrated potential to degrade plastic, although concerns exist regarding biomass safety due to a possible transfer of microplastic residues into food chains [102].

While supporting roles are often seen as background functions, they underpin agroecosystem stability and sustainability. Many insects involved in soil formation, nutrient cycling, and microbial community processes also participate in insect-based provisioning systems as sources of feed, food, and/or biofertilizers. This functional overlap—which occurs with *H. illucens* and *M. domestica*, and which contributes to both nutrient cycling and biomass production—blurs traditional ecosystem service categories, highlighting the need for integrated management perspectives. Supporting roles not only provide isolated contributions but also generate feedback loops with regulatory and provisioning functions, amplifying both benefits and risks. As products of insect bioconversion may re-enter the food system, concerns such as allergenicity, toxin bioaccumulation, and microbial hazards must be addressed. Recognising the centrality of these often-overlooked supporting functions is essential for designing insect management strategies that are truly systemic and context-responsive.

## 4. Towards Holistic Insect Management: Key Areas Necessary for Decision-Making

**Figure 2:**
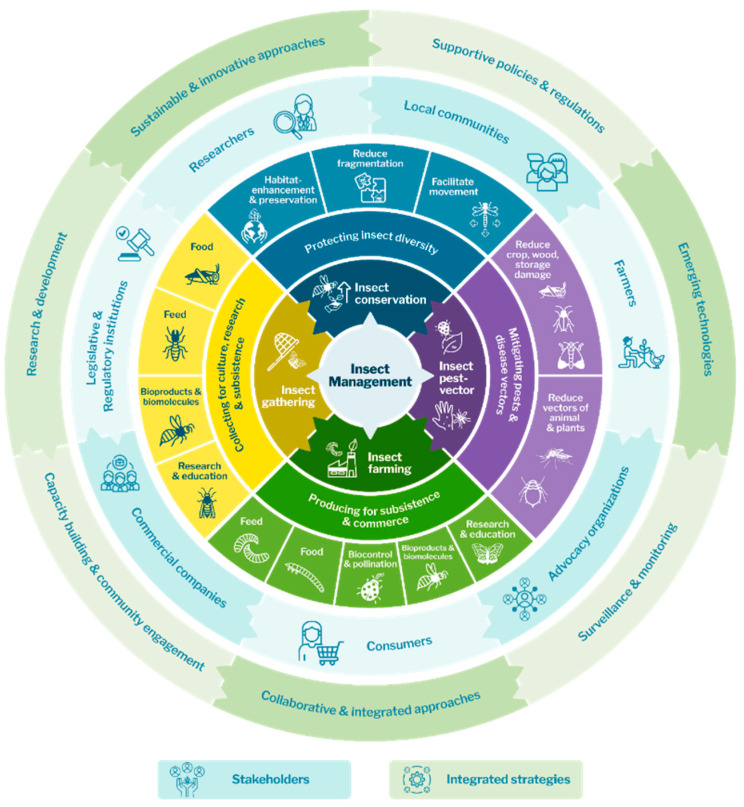
Integrated framework for promoting agrifood sustainability through effective insect management.

While different insect management strategies may each have different objectives, they often involve similar stakeholders. These include individuals and communities whose livelihoods directly depend on insects for food, income, and/or ecological services, as well as those impacted by the negative effects of insects as pests or disease vectors. Effective insect management decision-making requires integrating both scientific and empirical knowledge into complex social and ecological systems. As previously suggested (see [103]), wildlife management decision-making should involve defining goals, assessing available information, selecting from alternative actions, and monitoring outcomes to evaluate effectiveness. Insect management may include a range of areas, including the conservation of beneficial insects, the control of pest and vector species, responsible wild insect collection, and sustainable insect farming. While the goals of these strategies may differ, ranging from biodiversity preservation to food security to disease control, they are not mutually exclusive. In fact, their alignment provides opportunities for synergistic, holistic approaches to insect management that simultaneously address ecological, social, and economic challenges (Figure 2).

Optimizing insect SER requires an integrative management approach based on three interdependent components: (1) identifying and engaging relevant stakeholders and strategies; (2) combining different management approaches; and (3) addressing regulatory challenges and leveraging opportunities.

The first component of integrative insect management—identifying and engaging relevant stakeholders—involves local communities, farmers, researchers, authorities, consumers, and private companies in designing context-specific strategies that reflect diverse needs and values, which is necessary to foster long-term ecological, economic, sociocultural sustainability. Integrating both scientific and traditional knowledge systems into insect management is essential to accounting for plurality of values, cultural perceptions, and the uses of insects throughout the world [17,60,104].

The second component of integrative insect management—combining different insect management approaches—allows for generating synergistic benefits. For instance, designing insect farming according to clear ecological and social criteria may simultaneously support conservation goals and vector control [52,105]. However, such combinations require intersectoral planning to avoid conflicts. For example, in some cases, pest eradication programs prohibit use of certain species for food or feed despite their nutritional potential in order to prevent their propagation, while other initiatives simultaneously seek to promote their use to achieve sustainability or food security [106,107]. This highlights the need to reconceptualise the dichotomous perception of insects as either pests or resources, rather promoting integrated perspectives aligned with sustainability and One Health objectives [37].

With respect to the third component of integrative insect management—addressing regulatory challenges and leveraging opportunities, regulatory frameworks must be updated to explicitly include insects in agricultural, health, environmental, and food safety legislation. Many regulations still prioritize vertebrates while overlooking arthropods, leading to internally inconsistent regulatory frameworks [106]. By contrast, recent regulatory advances—such as those in the European Union recognizing insects as feed and food—indicate the potential for advocacy and coordinated efforts to shift policy [108]. Moreover, scaling local initiatives—for example, insect-peacebuilding projects in Colombia and insect-based circular economies in Africa—by adapting them to new contexts may contribute to more inclusive, place-based insect management strategies [61,109,110].

This multidimensional perspective reveals the limitations of treating insect conservation, pest control, gathering from the wild, and farming as separate agendas. In many contexts, these strategies fall under distinct institutional mandates, leading to inconsistent regulation and contradictions among policies, while overlooking synergies. Given that control of a particular pest species may be necessary in one setting, while the same species may serve as a valuable food source or cultural asset in another, there is a need for better coordinated, context-sensitive management approaches to avoid contradictions among strategies. Additionally, insects central to local diets or traditions may remain invisible to conservation policies that prioritize vertebrates and charismatic species. Such inconsistencies reduce the effectiveness of each strategy and hinder efforts systemically take into account the socio-ecological roles that insects fulfil.

Given that insects simultaneously influence ecosystems as well as human systems including agriculture, health, and culture, their management requires coordinated approaches. The SER framework provides a lens through which to identify points of convergence among these systems and support decision-making aligned with sustainability and equity. Rather than addressing insect-related issues through isolated initiatives, we propose a regulatory framework that combines four key areas of insect management—conservation, pest and vector control, wild insect gathering, and insect farming—as complementary tools within a broader system. The following sections examine each of these areas, illustrating how they may work in synergy to optimize insect contributions and mitigate risks.

### 4.1. Insect Conservation Management

Conservation strategies are aimed at preserving insect diversity and ecosystem functions by protecting habitats, mitigating threats such as pesticide use and climate change, and enhancing landscape connectivity. These actions reinforce supporting and regulating SER, including pollination, pest control, nutrient cycling, and soil formation, which are increasingly threatened by land-use change and other anthropogenic disturbances [111,112,113]. Nonetheless, conservation efforts are often designed in isolation, typically by environmental agencies, disconnected from agricultural and food policy [106,110]. Such fragmentation undermines potential synergies; for instance, while protected areas may exist for pollinators, adjacent cropland frequently relies on intensive agrochemical use, limiting insect conservation.

As many insect-mediated SER depend on functional landscapes in which environmental conservation goals and agricultural production needs intersect, it is essential to transcend sectoral divisions (e.g., agriculture, environment, health), rather implementing integrative frameworks that support both objectives simultaneously. A more inclusive approach to conservation management would incorporate agricultural actors—particularly farmers—not merely as beneficiaries of conservation efforts but as active agents in decision-making regarding legislation. Pollinator-friendly practices such as the establishment of hedgerows and flower strips, as well as pesticide reduction, benefit both biodiversity and yield [114]. Such synergies are particularly relevant in the Global South, where smallholders heavily depend on ecosystem services while facing pressure to intensify. Moreover, conservation must extend beyond charismatic or well-known species. Functionally crucial yet less visible taxa such as dung beetles, decomposers, and parasitoid wasps play vital agroecosystem roles. Culturally significant insects also require habitat protection to ensure continuity of traditional uses in medicine, food, and rituals [10,52]. In this context, even insect farms—particularly those based on native species and local substrates—can reduce pressure on wild populations while also generating alternative livelihood options for local communities [60,61,104]. However, potential risks such as the escape of exotic species and genetic homogenization must be considered, particularly near sensitive ecosystems [37]. Systemic insect conservation requires transforming landscapes through concrete local initiatives such as participatory monitoring, as well as through transdisciplinary initiatives that bring together science, policy, and traditional knowledge [17,115,116] and align biodiversity conservation with agri-food sustainability and biocultural resilience [117].

### 4.2. Insect Pest and Vector Management

Many insect species for which disruptive effects—including damaging crops, spreading pathogens, and undermining food systems—have been described above require targeted management approaches. However, rather than viewing pest and vector control in isolation, such approaches should be reframed as key to integrated insect governance. Mismanagement not only weakens regulating services such as natural pest control and pollination but also increases vulnerability in already fragile systems. A systemic, contextualized approach to pest and vector control allows for reducing risks that may escalate, affecting interconnected systems, while also providing opportunities for food security, One Health synergies, and ecological balance.

Conventional insect management approaches have often prioritized their eradication using chemicals, inadvertently harming beneficial insects while promoting pesticide resistance in pest species. Such methods conflict with the goal of safeguarding ecosystem services. Integrated pest management (IPM) provides an alternative management approach which combines ecological and biological techniques with cultural knowledge in order to minimize chemical use. Agroecological techniques such as intercropping and push–pull systems (which combine repellent and attractant plants to divert pests) allow for aligning pest control with sustainability [111,112]. Additionally, native predators and parasitoids may help regulate pest populations without harming pollinators or soil organisms. However, insect management becomes more complex in regions where certain pest species also hold nutritional and/or cultural value. For instance, *S. gregaria* and *Rhynchophorus* spp. are conventionally considered pests, yet are traditionally consumed locally in many regions [52,106,107]. This dual status poses regulatory dilemmas; should these species be eradicated, controlled, or sustainably managed as resources? The controlled harvesting of such insects may reduce crop loss while generating food and income. Given the apparently contrasting roles of edible insect pests, there is a need for specific regulation to ensure safety, define legal uses, and prevent undermining those actions aimed at controlling agricultural damage and population outbreaks. Taking into account such multifunctionality allows for bridging pest management with both food systems and cultural practices.

Innovative biotechnological strategies using insects may contribute to pest population regulation. For example, releasing sterile males of pests such as some dipterans reduces their populations without releasing chemicals into the environment [118]. Other approaches to biological control include releasing natural enemies such as *Trichogramma* wasps to suppress egg-laying and control larvae development [36]. Thus, insect husbandry extends beyond provisioning to contribute to ecosystem regulation.

Coherence among policies is critical to achieving sustainable insect management. In some regions, regulations ban the use of certain pest species in food and feed systems, despite their nutritional potential and cultural relevance, with the supposed goal of protecting public health or preventing the spread of disease. Nevertheless, this may undermine opportunities for sustainable use and community engagement. Therefore, there is a need for integrated governance that facilitates multifunctional use of insects within clearly defined ecological and safety boundaries [106]. By conceiving of pest and vector control as existing within a broader framework of socio-ecological roles, management strategies may transcend reactive suppression, rather becoming transformative tools to reduce harm to ecosystems and communities, create value, and increase the resilience of agri-food systems.

### 4.3. Wild Insect Gathering Management

Wild insect gathering is a longstanding practice that contributes to the provisioning of nutrient-dense food, as well as to local economies, particularly in Indigenous, rural, and low-income communities [14,119]. This strategy consists of collecting insects of various life stages from larva to adult for consumption, medicinal purposes, rituals, and commercial use. Nearly 56% of edible insect species are consumed as larvae (e.g., beetles), while 44% are consumed as adults (e.g., grasshoppers) [120,121,122]. Insect consumption contributes to dietary diversity and food sovereignty, provides income opportunities, and preserves traditional ecological knowledge, cultural and symbolic practices deeply embedded in local cosmovisions, and local biodiversity stewardship [123,124].

A wide variety of species—including *R. palmarum*, *Polistes* spp., termites, beetles, and Lepidopteran larvae—remain central to the diets and traditions of many communities throughout Latin America, Africa, and Asia [119,120,124,125]. However, fragmented regulations often place these insects in legal “limbo”; as neither wildlife nor agricultural products, they fall outside formal regulatory structures, leaving the practice of insect consumption vulnerable to both criminalization and neglect [106].

This regulatory invisibility presents challenges. Health risks linked to allergenic compounds, pathogens, parasites, and chemical contamination are rarely monitored, and due to anti-nutritional factors, insects may require careful preparation to ensure safety [126]. At the same time, given that wild insect gathering requires minimal inputs, its simplicity and accessibility make it vital to marginalized populations [54,117], underscoring the need for coherent policies that balance health protection with cultural practices and food sovereignty [120,123].

Rather than viewing wild gathering as a substitute for insect farming or conservation, it should be understood as a complementary strategy that may support functional diversity. Many insects which are currently farmed—including *H. illucens*, *T. molitor*, and *Z. atratus*—were first collected in the wild. Others which cannot be reared ex situ require sustainable in situ harvest [124]. Wild collection thus contributes to their scientific identification and prioritization of species for farming or conservation, informs conservation priorities, and supports ecological functions in different types of landscapes.

Integrated insect harvesting strategies, such as managing aquatic ecosystems for Hemiptera egg harvest and enhancing host plant availability for larvae for beetles which are traditionally gathered, demonstrate that ecological knowledge may contribute to optimizing yields. While these community-based strategies and initiatives may be context-specific, they illustrate how wild insect gathering may support peacebuilding, cultural revitalization, and circular economies [61,127]. For example, in some regions wild insect gathering is integrated into ecotourism and biodiversity education programs [21,109].

To fully unleash the SER potential of wild insect gathering, it must be formally recognized within policy and resource management planning frameworks. This includes developing sustainable harvest protocols, establishing food safety guidelines, and promoting participatory monitoring. These measures not only improve safety and sustainability but also help position wild insect gathering as a multifunctional strategy that reinforces biocultural resilience and strengthens ecosystem functionality within holistic insect regulatory frameworks.

### 4.4. Insect Farming Management

Insect farming has emerged as a strategic management approach to enhancing key insect SER—particularly provisioning and regulating services. Originally developed to domesticate insects with socio-economic importance [124], insect husbandry has rapidly expanded in recent years to contribute to animal feed, human food, waste bioconversion, biocontrol, and research. This reflects the growing recognition of insects’ high feed conversion efficiency, low environmental footprint, and capacity to decompose organic matter, all of which have been previously discussed under other SER categories [108,127].

Commonly farmed species, including *H. illucens*, *T. molitor*, and *A. domesticus*, are currently produced on various scales on several continents [127]. In addition to insects being used for biomass production, their by-products—including frass—are increasingly applied as fertilizer, promoting nutrient cycling and reducing reliance on synthetic inputs [128]. Thus, insect farming could be promoted as a strategy for reinforcing regulating ecosystem services and supporting functional biodiversity, which may contribute to establishing circular economies in which insects transform low-value organic matter such as food waste and manure into high-value outputs, including protein-rich biomass and biofertilizer [129].

Despite these benefits, the expansion of insect farming requires addressing significant biosafety and ecological risks. Microbial contamination, allergenicity, and chemical accumulation in larvae fed on inadequately processed substrates pose public health concerns [130]. Large-scale farms located near sensitive ecosystems poses the risk of escape of exotic species, competition with native decomposers, and the genetic homogenization of wild populations [110]. To ensure that benefits outweigh potential trade-offs, a One Health perspective is essential to guiding decisions regarding choice of substrates, containment systems, and monitoring standards [37].

In addition to addressing environmental and health considerations, insect farming must be understood as a socially embedded practice involving diverse actors, including researchers, producers, regulators, and communities, all of whom much be jointly engaged in decision to provide legitimacy to policies and contribute to resilience of insect farming system. Participatory research, capacity building and technical training, and inclusive regulatory mechanisms foster trust, promote the adaptation of practices to local contexts, and strengthen accountability by producers as well as regulatory agencies regarding environmental, public health, and social outcomes [17,37,130]. Successful examples of inclusive insect farming include decentralized production in Africa and Latin America that prioritizes native species, values local knowledge, and supports rural development and peacebuilding [61,104].

Insect farming is functionally interconnected with other management areas, including wild insect gathering and pest control, for example, given that several species currently farmed are collected from the wild, and that in some regions, semi-domesticated systems still operate alongside commercial farms. Moreover, insect rearing is essential to pest and vector control programs involving release of sterile insects [49]. Such interconnections between insect farming and other insect management areas indicate that insect husbandry may support multiple SER in different contexts. In recent years, policy frameworks regarding insect farming have begun to be adapted to reflect sectoral growth. The European Union has established regulatory guidelines for using insects as feed and food, largely through coordinated efforts by international and regional alliances and organizations including the International Platform of Insects for Food and Feed (IPIFF), the European Food Safety Authority (EFSA), the Academic Society for Insects as Food and Feed (ASIFF), the Asian Food and Feed Insect Association (AFFIA), and the Insect Network of the Latin American Association of Animal Production (ALPA) [119]. However, global disparities persist with respect to biosafety standards, substrate regulation, and traceability protocols. Closing these gaps will be crucial to safely and equitably scaling insect farming. To fully harness insects’ SER, insect farming must move beyond a purely productive paradigm. A systems-oriented, context-sensitive approach rooted in One Health principles will enable insect husbandry to function not just as a tool for producing protein, but also as a platform for ecological restoration, circular economies, and social inclusion

## 5. Optimizing Insect Socio-Ecological Roles: Synergies, Management, and Future Directions

Although insects play essential ecosystem roles, their contributions continue to be underrepresented in ecosystem service assessments, many of which emphasize few insect roles—including pollination, pest control, and organic waste recycling—and rely on general proxies such as species richness or abundance, rather than linking services to specific species or management contexts [131]. This limited perspective reinforces taxonomic and functional biases and overlooks the broader spectrum of roles that insects fulfil in sustainability and human well-being [131,132]. Recent work has also highlighted the need to develop specific indicators to measure these contributions in contexts such as insect farming, framing them within the One Health approach to better capture both benefits and risks [133].

In practice, insect contributions are far more diverse and context dependent. Globally, pollinators alone contribute an annual estimated USD 235–577 billion to agricultural production [134,135], and in the United States the combined services of insects—including pollination, pest control, animal dung burial, and ecotourism—are valued at over USD 57 billion annually [21]. However, these estimates often remain fragmented and fail to account for the multifunctionality of insects within cultural landscapes, local food systems, and ecological restoration [136].

Addressing this gap requires integrative approaches that do not address insect management strategies in isolation, but rather analyse conservation, pest and vector control, wild insect gathering, and insect farming as interconnected responses to local ecological, economic, and social dynamics [110]. Given that insects may play multiple roles in these strategies—as pests, food, decomposers, and/or pollinators—there is a need to develop synergistic context-sensitive insect management models [19]. The four insect management areas presented above reflect the diversity of insect management practices and their contributions to ecosystem services, sustainability, and human well-being. However, their full potential may emerge when all four are applied in combination. Table 1 summarizes each area and illustrates how they may be combined to achieve integrated initiatives. Such examples highlight the importance of holistic insect governance frameworks that incorporate diverse strategies, stakeholders, and knowledge systems.

A recent exploratory study [19] proposes a multidimensional framework by which experts evaluated 120 insect species based on their based on their productive potential, ecosystem roles, contextual use, and associated challenges. The findings illustrate how assessing species in this way can inform evidence-based decision-making, for example, by identifying native species with high productive and/or ecological value that could be prioritized in farming, and by recognizing overlooked insects that play key cultural and/or environmental roles in specific territories. Embedding such evaluations into management planning may provide a practical basis for linking species’ socio-ecological roles with targeted actions across different management areas.

While many innovative initiatives have combined more than one management area—such as insect farming coupled with wild gathering or conservation integrated with pest management—they typically involve only two such areas and remain largely disconnected from broader governance frameworks. Optimizing the socio-ecological roles of insects requires inter- and transdisciplinary approaches to insect management that incorporate diverse knowledge systems and foster collaborative planning. Scientific research must be coupled with dialogue among stakeholders to design management strategies that reflect both biodiversity goals and societal needs. Grounding insect-related regulatory frameworks in systemic thinking and contextual evidence may allow for moving beyond fragmented interventions toward resilient agri-food systems that embrace insects as agents of many types of sustainability.

## Figures and Tables

**Table 1:** Strategic approaches to insect management and their integration into agri-food systems taking advantage of insects’ socio-ecological roles.

Management Area	Main Objective	Associated Ecosystem Services	Associated Problems	Key Stakeholders	Integration Needs	Example of Cross-Area Synergy
Insect conservation	Preserve native insect diversity and ecosystem functions.	Pollination and seed dispersal (regulation); cultural symbolism (cultural service); bioindication (regulation); soil regeneration (support).	Decrease in pollinators; entomophobia and stigma; invasive species replacing native species.	Academics, farmers, activists, policymakers, researchers, educators.	Link biodiversity protection to agri-food systems; promote culturally sensitive conservation.	Conservation of native dung beetles supports soil regeneration and may inform species selection for insect farming.
Insect pest and vector management	Reduce crop losses and disease transmission by managing pest/vector species.	Biological control (regulation); vector monitoring (regulation).	Pesticide resistance; ecological imbalance; health impacts.	Farmers, agroecologists, health authorities, policymakers, extension workers.	Combine monitoring with agroecological control; incorporate local knowledge.	Wild gathering of pest species can provide food and help reduce outbreaks.
Wild insect gathering	Sustain local livelihoods and traditional insect uses through responsible harvesting.	Food/feed provision (provisioning); cultural heritage (cultural service); biomass cycling (support).	Overharvesting; lack of safety standards; criminalization.	Indigenous groups, other harvesters, policymakers, local markets, NGOs.	Develop biocultural protocols; support community-based monitoring; ensure legal recognition.	Wild gathering of farmed species can help protect genetic diversity and prevent disease outbreaks in insect farming.
Insect farming	Produce insects for food, feed, waste reduction, biocontrol and bio-products.	Organic matter conversion (support); protein/fertilizer production (provisioning); circular innovation (support).	Contamination; limited regulation; low species diversity.	Farmers, researchers, regulators, policymakers, investors.	Improve biosafety norms; regulate inputs; diversify species.	Insect farming based on native biodiversity may promote ecosystem restoration and community income.

## Data Availability

No new data were created or analyzed in this study. Data sharing is not applicable to this article.

## Abbreviations

The following abbreviations are used in this manuscript:

ES	Ecosystem Services
SER	Socio Ecological Roles
MEA	Millennium Ecosystem Assessment
SDG	Sustainable Development Goals
IPM	Integrated pest management
NGO	Non-Governmental Organization.
PM	Particulate Matter
IPIFF	International Platform of Insects for Food and Feed
EFSA	European Food Safety Authority
ASIFF	Academic Society for Insects as Food and Feed
AFFIA	Asian Food and Feed Insect Association
ALPA	Latin American Association of Animal Production
